# Case Report of a Saga of Post-COVID-19 Complications

**DOI:** 10.7759/cureus.16247

**Published:** 2021-07-07

**Authors:** Sangita D Kamath, Bharti Sharma, Jayanta K Laik, Manish Kumar, Ashok Sunder

**Affiliations:** 1 Internal Medicine, Tata Main Hospital, Jamshedpur, IND; 2 Ophthalmology, Tata Main Hospital, Jamshedpur, IND; 3 Joint Replacement and Orthopedics, Tata Main Hospital, Jamshedpur, IND

**Keywords:** covid-19, thrombosis, arthritis, empyema, endophthalmitis

## Abstract

Coronavirus disease 2019 (COVID­-19), caused by severe acute respiratory syndrome coronavirus 2, is characterized by symptoms such as fever, sore throat, cough, fatigue, myalgias, headache, diarrhea, and dysgeusia. In a majority of the cases, it causes mild illness. However, in severe cases, the virus activates the immune system causing systemic inflammation, immune dysregulation, and pro-thrombotic state leading to various complications. Among hospitalized patients with COVID-19, pneumonia, sepsis, and respiratory failure are frequent complications. However, even in the second year of the COVID-19 pandemic, our knowledge of its myriad clinical features and complications is still incomplete and continues to evolve. Here, we present the case of a patient who developed several complications post-COVID-19 one after the other. He was admitted with severe COVID-19 for which he received standard COVID-19 treatment and mechanical ventilation. In the post-COVID-19 state extending up to six months, he serially developed deep vein thrombosis, endogenous endophthalmitis, empyema, and post-inflammatory arthritis of the hip joints. To our knowledge, such a case has not been reported earlier in the literature.

## Introduction

The coronavirus disease 2019 (COVID-19) pandemic, caused by the severe acute respiratory syndrome coronavirus-2 (SARS-CoV-2) infection, reportedly started via zoonotic transmission in the Wuhan province of China and spread globally with almost no country remaining unaffected. The World Health Organization declared the outbreak a pandemic on March 11, 2020 [1]. Not much is known about the natural history of SARS-CoV-2 infection. The respiratory illness caused by this virus was termed coronavirus disease 2019. The disease is transmitted via infected respiratory droplets and aerosols. It has diverse clinical symptoms involving almost all organ systems including the lungs, liver, heart, kidney, and nervous system but predominantly affecting the respiratory system. The mortality rate of SARS-CoV-2 infection signiﬁcantly varies globally, ranging from 0.3% to 8.4% [2].

While most patients recover without complications (80%), few develop complications with a life-long impact on their health. A dysregulated hyperimmune response resulting in cytokine release storm is responsible for severe cases and organ damage [2]. SARS-CoV-2 targets angiotensin-converting enzyme 2 (ACE2) receptors to infect host cells. High expression of ACE2 receptors in the lung tissue can explain the predominance of lung involvement. The spectrum of complications and sequelae following COVID-19 infection continues to evolve as more and more literature is reported. Here, we report the case of a patient who unfortunately suffered from a myriad of complications following COVID-19 infection.

## Case presentation

**Figure 1:**
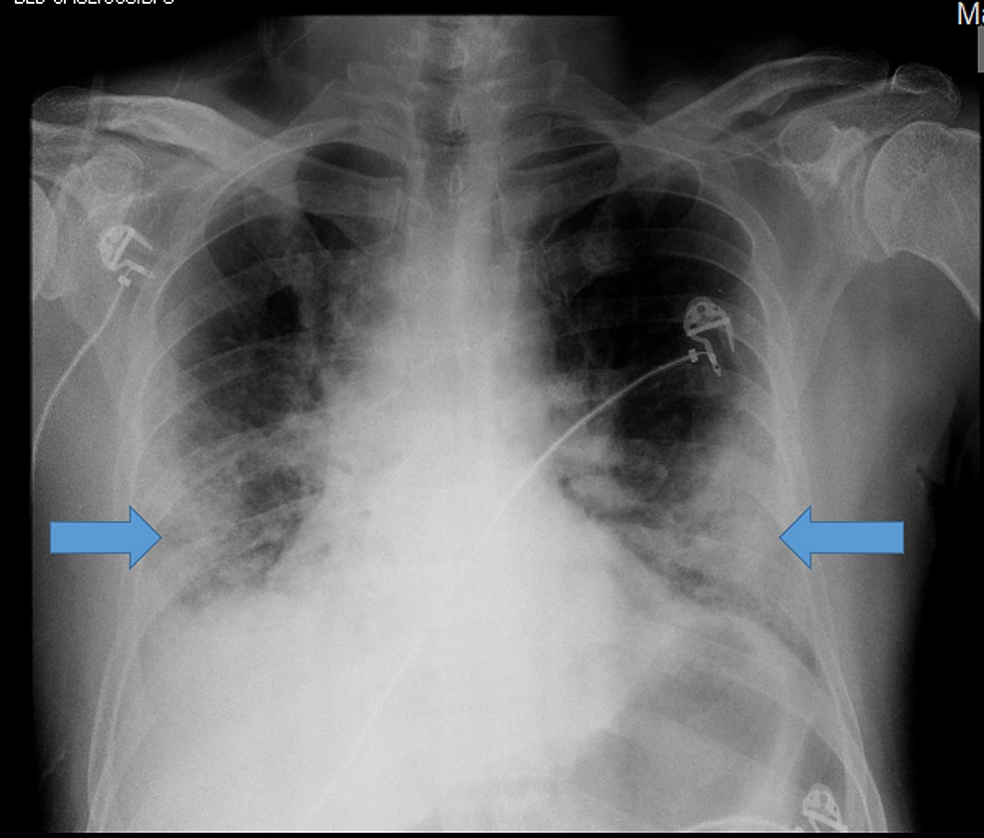
Chest radiograph showing bilateral peripheral opacities in mid and lower zones.

A 49-year-old gentleman with a medical history of type 2 diabetes mellitus with diabetic retinopathy, hypertension, and hypothyroidism presented to the emergency department with complaints of fever, cough, and breathlessness for five days. His chest X-ray revealed bilateral lower zone consolidations (Figure 1), and throat swab reverse-transcription polymerase chain reaction (RT-PCR) for SARS-CoV-2 was positive.

**Figure 2:**
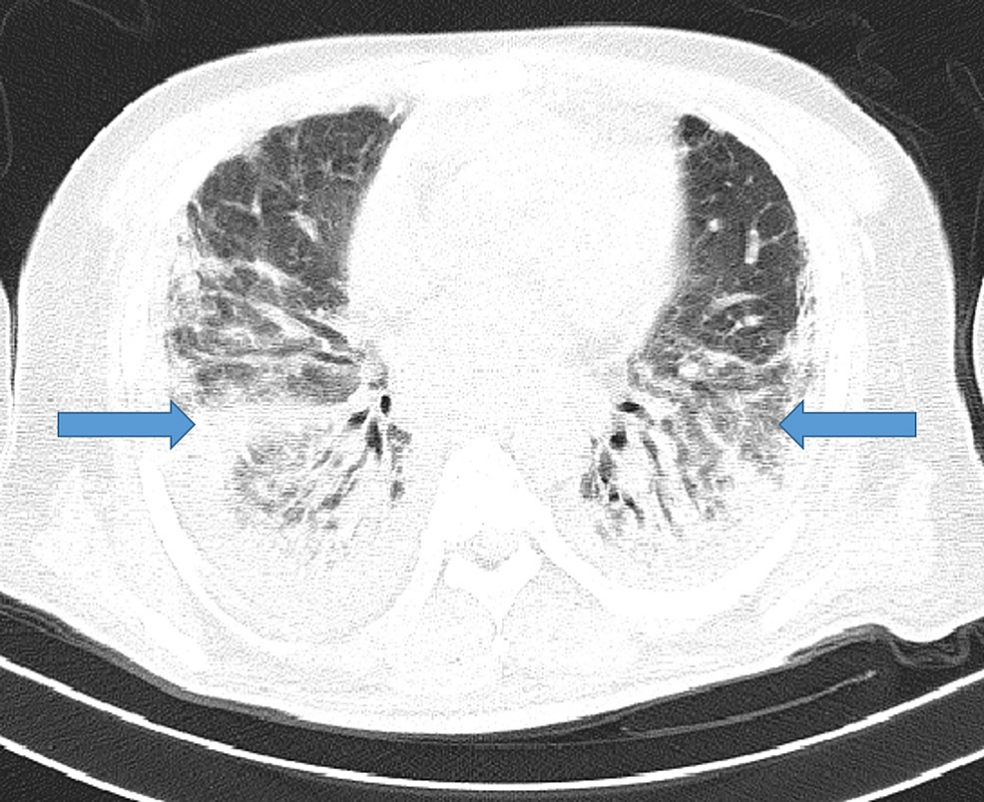
HRCT of the thorax showing bilateral ground-glass opacities and consolidation. HRCT: high-resolution computed tomography

A high-resolution computerized tomography (HRCT) of the thorax done two days after hospital admission showed bilateral scattered ground-glass opacities in all lobes, areas of focal consolidations, and bilateral mild pleural effusions (Figure 2). His CT severity score (CTSS) was 18/25 with the COVID-19 Reporting and Data System (CO-RADS) scale of 6.

The patient turned hypoxic and was transferred to the critical care unit (CCU) where he received protocolized therapy for COVID-19 in the form of intravenous remdesivir, steroid (methylprednisolone 32 mg twice a day intravenously), convalescent plasma, cefepime 1 g twice a day for seven days intravenously, subcutaneous low-molecular-weight heparin (LMWH) (enoxaparin 60 mg twice a day), high-flow nasal oxygen at 60 L/minute, and non-invasive ventilation. In view of persistent hypoxemia, he was intubated and placed on mechanical ventilatory support (with initial FiO_2_ of 90% and positive end-expiratory pressure of 10 cm H_2_O) three weeks after admission. As his inflammatory markers (serum ferritin, C-reactive protein, and interleukin-6) were high, he also received tocilizumab 400 mg intravenously, a single dose in view of the cytokine storm. He was tracheostomized on the seventh day of intubation. He was gradually weaned off ventilator support over the next 15 days, his tracheostomy was decannulated, and he was subsequently shifted out of the CCU on oxygen support at 8 L/minute via a non-rebreathing mask to the High Dependency Unit (HDU).

**Figure 3:**
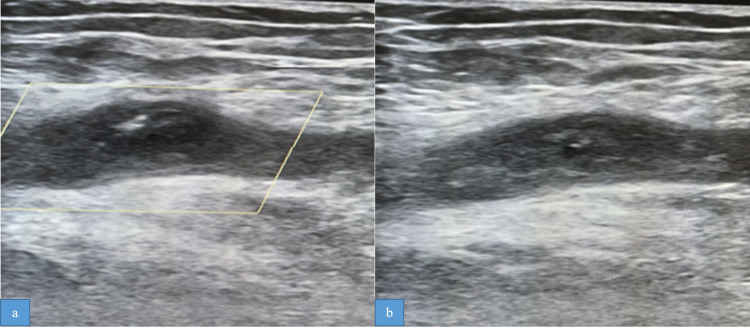
Doppler images. (a) Doppler study (gray scale) shows dilated non-compressible right common femoral vein with an echogenic intramural thrombus. (b) No color pick-up is seen on the color Doppler image.

In the HDU, he was noticed to have swelling of both lower limbs with disproportionate swelling of the right leg with pain in the thigh. An urgent Doppler study of the venous system in the legs showed deep vein thrombosis (DVT) in the right common femoral and proximal great saphenous vein (Figures 3a, 3b), while the Doppler study of the arterial system showed diffuse atherosclerotic changes with wall calcification in both legs.

His oral anticoagulation was upgraded to injection enoxaparin 60 mg twice daily subcutaneously for five days followed by oral apixaban 5 mg twice daily. Meanwhile, his chest radiograph showed partial clearing of the opacities, and his oxygen saturation (SpO_2_) on ambient air improved to 88%. He was discharged on domiciliary oxygen therapy, oral methylprednisolone 16 mg once a day, apixaban 5 mg twice a day, and oral anti-diabetic drugs. A repeat venous Doppler study of the right leg done after three weeks of starting anticoagulation therapy showed complete resolution of the thrombus.

**Figure 4:**
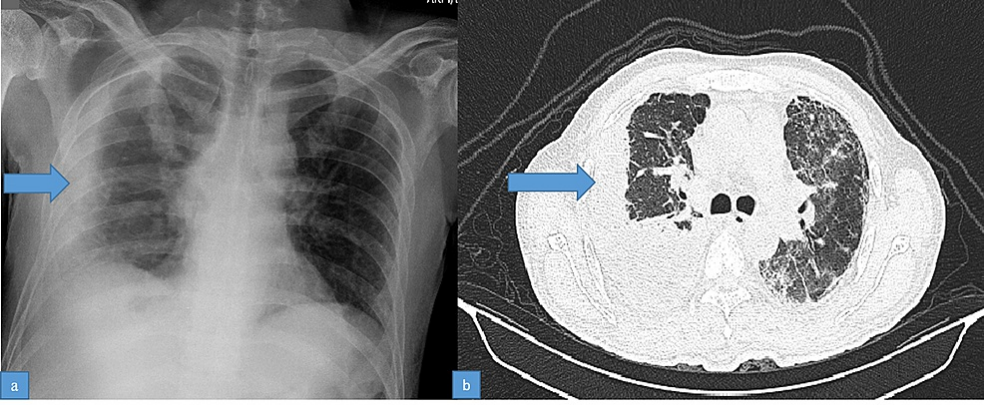
(a) Chest radiograph showing empyema on the right. (b) HRCT of the thorax showing right empyema. HRCT: high-resolution computed tomography

He was readmitted one week later with fever and breathlessness associated with pain and redness of the right eye. He was tachypneic with SpO_2_ on ambient air being 78%. His chest radiograph and HRCT of the thorax showed moderate right pleural effusion (Figures 4a, 4b).

Ultrasound of the chest showed loculated right pleural effusion, and it was tapped under radiological guidance. Fluid was turbid and exudative in nature. The total white blood cell count was 8,250/mm^3^ with 95% polymorphs suggestive of empyema. Adenosine deaminase level was 24.6 U/L. He was started empirically on intravenous meropenem 1 g TID and levofloxacin 750 mg OD and an intercostal drain was placed. Culture of the pleural fluid later revealed growth of *Pseudomonas aeruginosa* sensitive to meropenem and hence the same antibiotics were continued for 15 days. The drain was removed after 15 days when there was no significant fluid draining.

**Figure 5:**
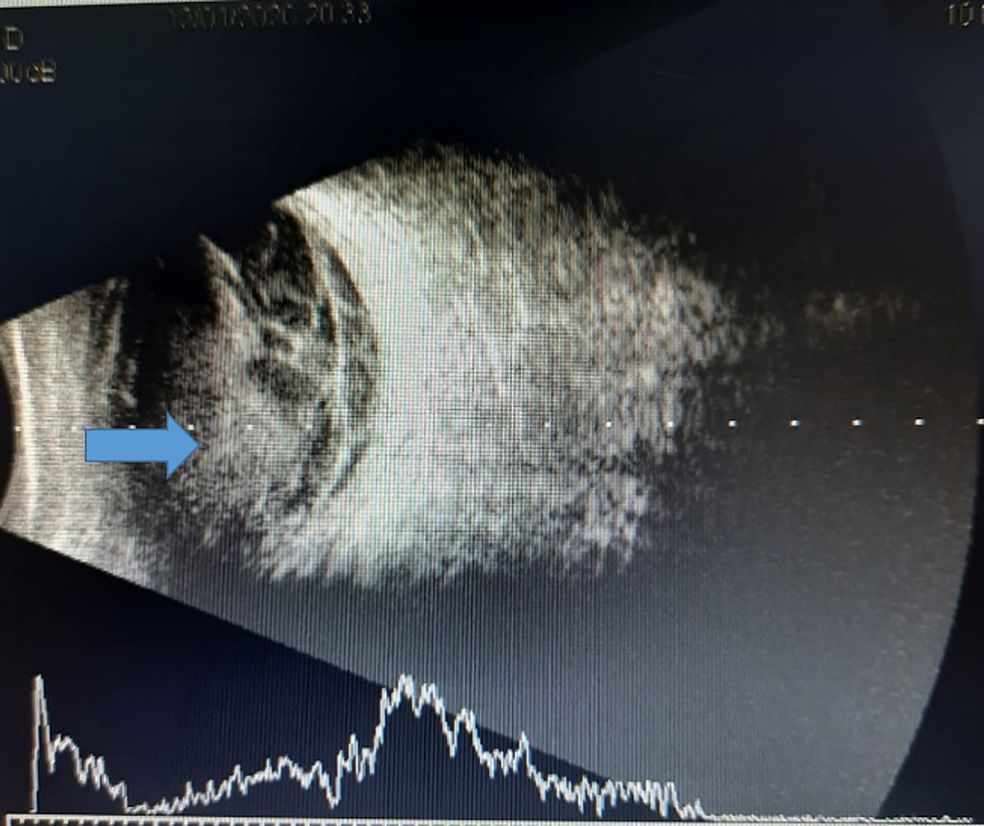
B-scan of the left eye showing multiple echoes in the vitreous cavity preoperatively suggestive of endophthalmitis.

On day four of the second admission, he developed diminution of vision in the right eye. On ocular examination, vision in the left eye was 6/6 while in the right eye he only had a perception of light. His intraocular pressure was 14 mmHg in both eyes. Left eye examination was unremarkable. Slit-lamp examination of the right eye revealed conjunctival congestion, clear cornea, 4 mm hypopyon, and posterior synechiae. B-scan showed low-intensity echoes in the vitreous and intact retina (Figure 5).

**Figure 6:**
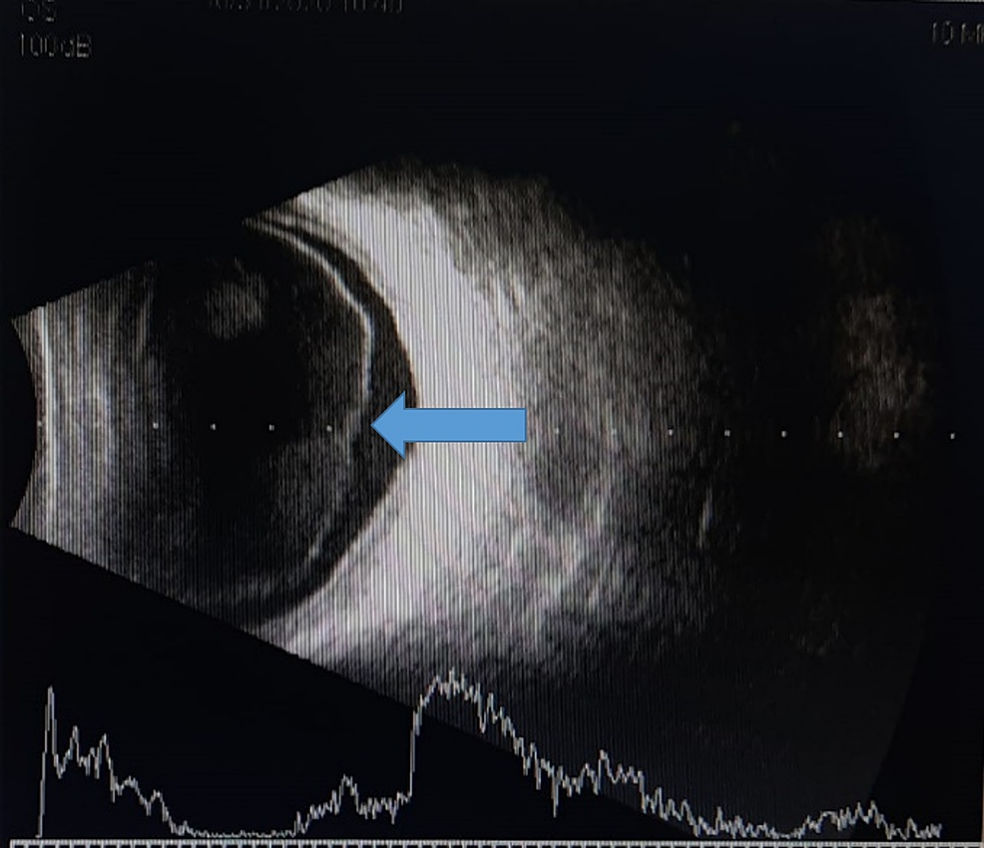
B-scan showing retinal detachment post-vitrectomy.

His blood culture showed growth of *Staphylococcus aureus*, while his urine culture was sterile. He was diagnosed with endogenous endophthalmitis and was started on topical ciprofloxacin and moxifloxacin eye drops every two hours along with homatropine and timolol eye drops. He also received intravenous ceftazidime 1 g thrice daily and vancomycin 1 g thrice daily. As his vitreous echoes increased, he was referred to a higher center, where he underwent urgent pars plana lensectomy and vitrectomy in his right eye. PCR from a vitreous sample from his right eye was positive for the eubacterial genome. He was given intravitreal injections of ceftazidime and vancomycin postoperatively for three days. After two weeks of the surgery, there was no perception of light in the right eye. On examination, his right eye had a clear cornea with aphakia. Fundus examination revealed total retinal detachment in the right eye. His right eye is quiet after six months of surgery with no perception of light and has total retinal detachment (Figure 6).

**Figure 7:**
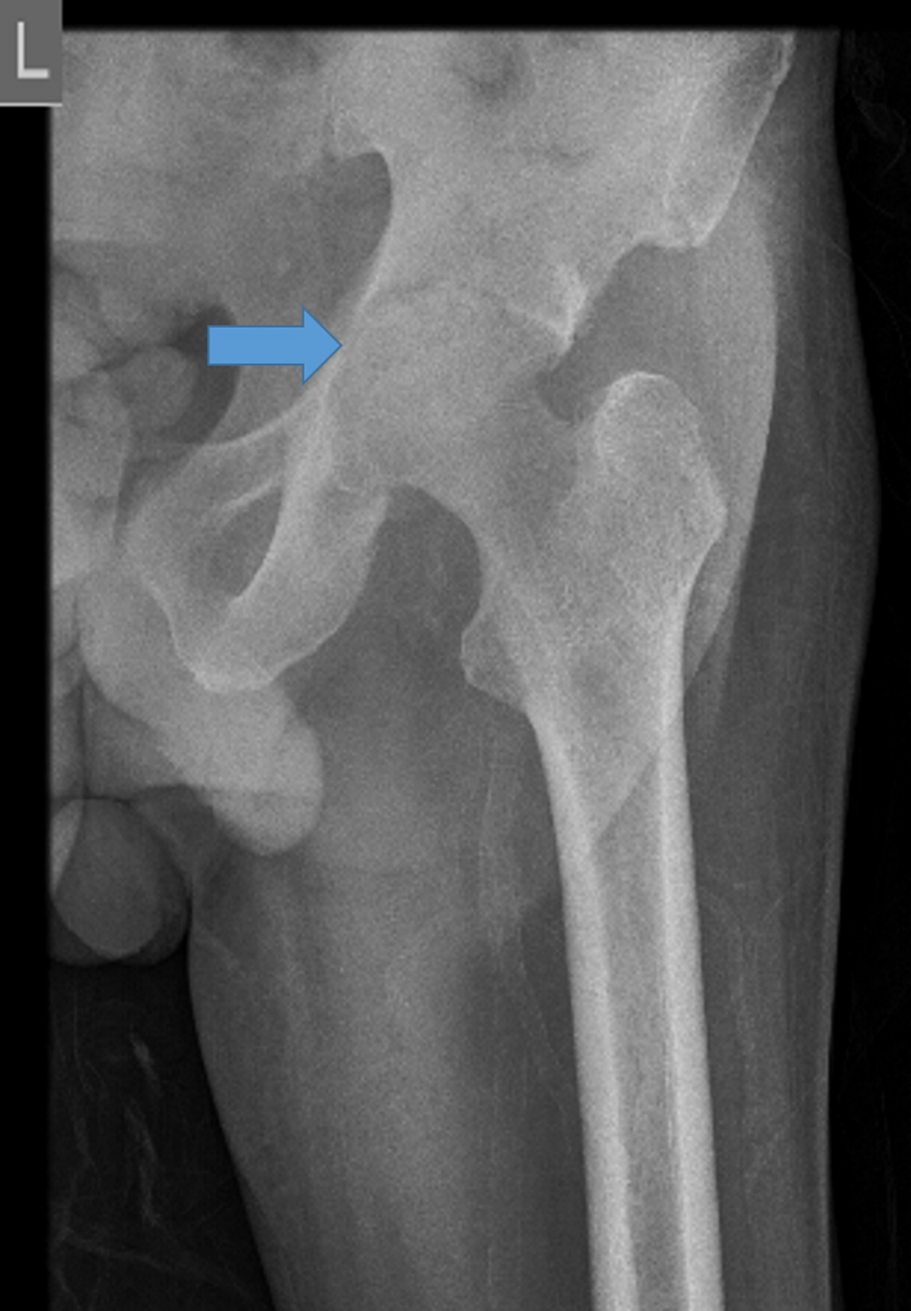
X-ray of the left hip joint showing reduced joint space.

He also complained of pain in the left hip joint which had started roughly 25 days after his first hospital admission. As the pain increased on stretching the leg, the patient would preferably keep his hip flexed. The pain increased progressively over four months and he was unable to bear weight and walk even a few steps due to pain. He had to move on a wheelchair to continue his activities of daily living. With these symptoms, he was re-admitted to our hospital five months after the index admission. There was no history of trauma, red eye, skin rash, and dysuria. On examination, there was tenderness over the left hip, and active straight leg raising test was not possible. Active toe and ankle movement were normal. A radiograph of the left hip showed decreased joint space suggestive of inflammatory reactive arthritis (Figure 7).

**Figure 8:**
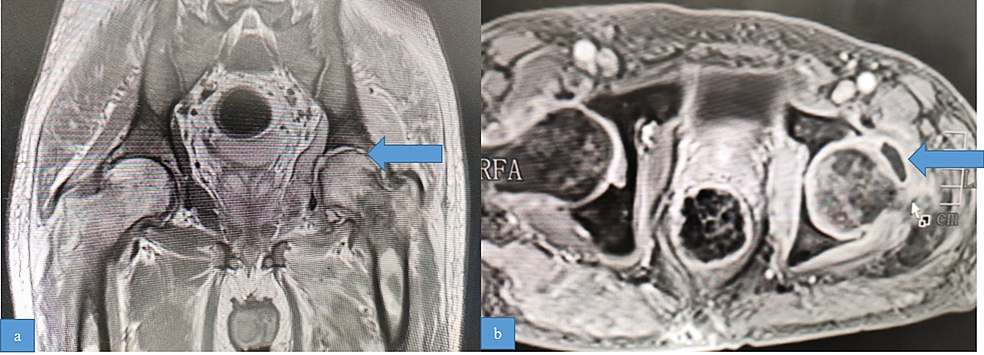
Post-contrast T1 FS sagittal and T1-weighted coronal images showing edema in the head and neck of the left femur, cortical irregularity of the head with loss of articular cartilage, and small joint effusion. FS: fat suppression; T1: time constant

The differential diagnosis considered were post-COVID-19 inflammatory arthritis, septic arthritis, tuberculosis of the left hip, and avascular necrosis (AVN) of the femoral neck. His tests for rheumatoid factor, anti-citrullinated peptide antibodies, and anti-nuclear antibodies were negative. His RT-PCR for SARS-CoV-2 was negative. Contrast-enhanced magnetic resonance imaging (CEMR) of the left hip showed hyperintensity of the left femoral head, neck, and acetabulum with post-contrast enhancement with effusion of the joint. There was mild hyperintensity in the right femoral head (Figure 8).

As there was bilateral involvement on MRI, the possibility of AVN or post-COVID-19 inflammatory arthritis was considered. Core decompression of the femoral head and neck was done through anterior approach mini-arthrotomy. A brownish-green fluid was drained. Smear and Gene-Xpert of the bone and synovial tissue and joint fluid did not reveal acid-fast bacilli. Cultures were also sterile. Histopathological examination of synovial tissue showed dense mixed inflammation comprising lymphocytes, plasma cells, histocytes, and a fair number of neutrophils with several dilated and congested blood vessels, while the bony tissue showed normal bone with hemopoietic elements. There was no evidence of granulomatous inflammation or malignancy, which was suggestive of non-specific inflammatory changes. He was treated with oral linezolid 600 mg twice daily and rifampicin 600 mg once daily for 15 days in addition to symptomatic treatment with non-steroidal anti-inflammatory drugs, following which pain in the hip joint subsided and he was able to bear weight and walk with support.

## Discussion

COVID-19, the disease caused by SARS-CoV-2, usually manifests as fever, sore throat, rhinitis, weakness, loose motions, and, rarely, loss of smell and taste, and a majority of the cases resolve without complications. However, in severe cases, the virus activates the immune system leading to systemic hyperinflammation and immune dysregulation leading to various complications which include acute respiratory distress syndrome, renal failure, myocarditis, cardiac arrhythmias, thrombotic events, stroke, sepsis, and multiorgan dysfunction [2]. As more cases are reported, it is seen that COVID-19 may result in complications involving almost every organ system. As this is a new virus, even in the second year of the COVID-19 pandemic, complications and the post-COVID-19 sequelae are not fully known and our knowledge continues to evolve. However, an exaggerated/deregulated inflammatory response is thought to be the culprit behind a majority of the complications. Even though complications involving various organ systems have been described in COVID-19, our case was unique in that multiple typical and atypical complications evolved with time in a single patient. Of the many complications seen in our patient, it is hard to know which to attribute to COVID-19. For example, whereas DVT and pneumonia are common in COVID-19, complications such as endophthalmitis, empyema, and inflammatory arthritis have been rarely reported in the literature and are not hitherto described in the same patient. We now discuss each of the complications that our patient unfortunately suffered.

Deep vein thrombosis

Our patient developed DVT of the right lower limb, which is a frequently reported complication in COVID-19 infection. COVID-19 infection is now known to be associated with a hypercoagulable state and thus is a specific risk factor for thromboembolism. The incidence of thrombotic complications is 16-49% among patients admitted to intensive care [3]. The pathogenesis of hypercoagulability probably involves endothelial injury as a result of the direct effect of the viral invasion, proinflammatory cytokines, and direct activation of the coagulation pathway by the virus. Endothelial cells are potential targets for SARS-CoV-2 because of the highly expressed ACE2 receptors. For those with severe COVID-19 requiring intensive care, a high risk of venous thromboembolism has been reported, despite thromboprophylaxis [4]. This was also seen in our patient who developed DVT despite being treated with LMWH as part of the COVID-19 protocol.

Septic endophthalmitis

Endogenous endophthalmitis is an intraocular infection caused by hematogenous spread from distant foci. Our patient presented within a week of being discharged, with symptoms of eye redness and pain along with fever and breathlessness. On investigation, he was found to have right-sided empyema. Evaluation of the eye revealed endogenous septic endophthalmitis. Despite aggressive management with intravenous and intravitreal antibiotics and surgery, his vision could not be saved. This highlights the aggressive nature of the illness and the importance of early recognition and treatment. Whether the endophthalmitis can be attributed to the preceding COVID-19 infection is unclear. It could be due to endogenous bacterial dissemination from the empyema or secondary to septicemia, as evidenced by the growth of *S. aureus* in blood culture.

Very few cases of endophthalmitis in COVID-19 have been reported. A case series by Bilgic et al. [5] reported three cases of endogenous endophthalmitis in patients recovering from COVID-19 pneumonia. Causative organisms included *Klebsiella pneumoniae*, *Stenotrophomonas maltophilia*, and methicillin-resistant *Staphylococcus aureus*. They also demonstrated the presence of SARS-CoV-2 by RT-PCR in the vitreous fluid of one patient. Another case series by Shah et al. [6] reported four cases of presumed fungal endophthalmitis wherein no organism could be isolated.

Empyema

Pleural complications observed in COVID-19 include pneumothorax, pneumomediastinum, and empyema. These can develop due to COVID-19-related pathophysiological changes, treatment interventions, or comorbidities of the patients. The most commonly reported complication is pneumothorax, while cases of empyema have also been reported. Ceylan et al. [7] reported four cases of empyema among 342 (1.2%) COVID-19 patients who were treated by tube thoracostomy and appropriate antibiotic therapy. In our case, combination of reactive pleural effusion and further exposure to risk factors like prolonged intubation and use of tocilizumab might have facilitated bacterial superinfection and consequent development of empyema.

Post-COVID-19 inflammatory arthritis

Our patient also suffered from bilateral asymmetric arthritis involving both hip joints. Important differentials considered included septic arthritis as the patient had suffered from other sepsis-related events such as endophthalmitis and empyema. However, the absence of fever, normal inflammatory markers, and sterile cultures ruled out septic arthritis. Besides, the temporal relation with SARS-CoV-2 infection made the hypothesis of post-viral inflammatory arthritis the most probable. Avascular necrosis of the femur was another differential in view of steroid use. However, MRI findings and histopathology helped rule it out. The musculoskeletal and rheumatic symptoms of COVID-19 (i.e., myalgia, fatigue, arthralgia) have been attributed to systemic immune response, pro-inflammatory cytokine release, and direct invasion/injury of musculoskeletal cells by SARS-CoV-2 through the ACE2 receptor [8].

Inflammatory reactive arthritis involving different joints has been reported as a complication occurring after COVID-19 infection from different parts of the world. *Lancet Rheumatology* reported presumably the first case of reactive arthritis in COVID-19 in Europe. The patient was a 58-year-old woman who presented with arthralgia involving the ankle 25 days after the COVID-19 infection. Ultrasound of the joint revealed synovial hypertrophy with inflammation of the Achilles tendon and the patient showed good response to NSAID therapy [9]. Mukarram et al. reported five patients who developed inflammatory arthritis following COVID-19 infection. All patients developed polyarthritis showing symmetrical distribution, including large and small (distal and proximal interphalangeal) joints, with musculoskeletal ultrasound scans showing evidence of symmetrical synovitis [10]. Ghauri et al. described a case of a 34-year-old male who developed reactive arthritis (right knee) 10 days after being diagnosed with COVID-19 [11]. Gasparotto et al. described right hip joint involvement along with the involvement of knee and ankle 13 days after COVID-19 infection. Arthrocentesis revealed the inflammatory nature of synovial fluid [12]. Progressive nature of the arthritis and absence of affection of joints other than the hip makes our case unique. Further studies are needed to understand the pathogenesis of arthritis in COVID-19.

The spectrum of post-COVID-19 sequelae is still not completely clear. As more and more literature is being reported, we are coming across rare and unusual complications. This was also the case in our patient who unfortunately suffered from multiple complications. This case report, therefore, aims to increase our awareness of the various complications which can be seen in COVID-19. This, in turn, may lead to the early recognition and more efficient management of COVID-19 patients with complications.

## Conclusions

We are still in the process of exploring the consequences of the novel SARS-CoV-2 infection. COVID-19 can cause a wide spectrum of sequelae. It induces a pro-coagulant state and can affect any organ including the eye, lungs, heart, and musculoskeletal system, though the respiratory system and lungs are commonly involved. Patients with severe COVID-19 show rapid advancement of the lung infiltrates and develop multiorgan dysfunction syndrome, related to the sustained release of inflammatory cytokines which typically occurs eight to ten days after the disease onset. However, few COVID-19 patients may develop a gamut of unusual complications with time related to ongoing immune system dysregulation and hence need a long-term follow-up.
